# Multimodal non-invasive non-pharmacological therapies for chronic pain: mechanisms and progress

**DOI:** 10.1186/s12916-023-03076-2

**Published:** 2023-09-29

**Authors:** Yu Shi, Wen Wu

**Affiliations:** Department of Rehabilitation, Zhujiang Hospital, Southern Medical University, Guangzhou, 510282 China

**Keywords:** Pain, Non-invasive non-pharmacological therapies, Physical modalities, Psychological interventions, Complementary and alternative therapies, TENS, CBT, TMS, VR

## Abstract

**Background:**

Chronic pain conditions impose significant burdens worldwide. Pharmacological treatments like opioids have limitations. Non-invasive non-pharmacological therapies (NINPT) encompass diverse interventions including physical, psychological, complementary and alternative approaches, and other innovative techniques that provide analgesic options for chronic pain without medications.

**Main body:**

This review elucidates the mechanisms of major NINPT modalities and synthesizes evidence for their clinical potential across chronic pain populations. NINPT leverages peripheral, spinal, and supraspinal mechanisms to restore normal pain processing and limit central sensitization. However, heterogeneity in treatment protocols and individual responses warrants optimization through precision medicine approaches.

**Conclusion:**

Future adoption of NINPT requires addressing limitations in standardization and accessibility as well as synergistic combination with emerging therapies. Overall, this review highlights the promise of NINPT as a valuable complementary option ready for integration into contemporary pain medicine paradigms to improve patient care and outcomes.

**Supplementary Information:**

The online version contains supplementary material available at 10.1186/s12916-023-03076-2.

## Background

Chronic pain conditions, such as osteoarthritis, diabetic neuropathy, and fibromyalgia, refer to a range of complex and multifaceted disorders that affect millions of individuals worldwide and impose significant burdens on society, healthcare systems, and personal well-being [1, 2]. The management of chronic pain presents a major challenge for healthcare professionals and researchers, as there is no single or universally effective treatment and patients often suffer from chronic pain for a long period of time [3, 4]. Furthermore, conventional treatments such as opioids and nonsteroidal anti-inflammatory drugs (NSAIDs) carry significant risks and limitations, including addiction, tolerance, dependence, gastrointestinal bleeding, renal impairment, and cardiovascular events, especially when used long term [5, 6]. As a result, additional approaches are needed to provide safe and effective chronic pain relief without causing harm or adverse effects [7].

Non-invasive non-pharmacological therapies (NINPT) offer options for managing chronic pain safely and effectively without damaging tissues or causing significant side effects [8]. For chronic pain conditions, NINPT can be categorized into physical modalities, psychological interventions, complementary and alternative therapies, and other innovative techniques [8, 9]. Physical modalities like transcutaneous electrical nerve stimulation (TENS), heat therapy and cryotherapy, and massage aim to modulate chronic pain signals through physiological mechanisms [10–12]. Psychological interventions including cognitive-behavioral therapy (CBT) and mindfulness-based therapy help patients cope with chronic pain by promoting positive psychological and behavioral changes [13–15]. Complementary and alternative therapies such as acupuncture, yoga, and music therapy provide additional approaches to reducing chronic pain through mechanisms involving both physical and psychological aspects [16–18]. Other interventions like chiropractic care, biofeedback, and virtual reality (VR) therapy employ techniques to modulate chronic pain [19–21]. For chronic pain management, NINPT can be used alone or combined with conventional treatments based on specific conditions, patient preferences, and availability of resources.

Furthermore, understanding the mechanisms underlying how NINPT alleviate chronic pain is critical for optimizing their clinical application. To begin with, it helps establish the causal relationship between NINPT and their effects on chronic pain, clarifying their validity and reliability [15, 22]. Secondly, elucidating the mechanisms of NINPT action allows for optimized, individualized therapeutic regimens meeting the needs of each patient. This includes determining the optimal frequency, intensity, and duration for achieving the best outcomes [23]. Thirdly, it spurs continued innovation in developing more effective and integrative NINPT modalities for chronic pain relief through emerging technologies [7, 24]. Lastly, establishing the neurophysiological mechanisms underlying the effects of NINPT on chronic pain enables the development of evidence-based clinical guidelines on recommending appropriate NINPT options for different chronic pain conditions [9, 25].

The objective of this review is to provide a comprehensive and up-to-date overview of various NINPT for chronic pain management, with a focus on their mechanisms of action and clinical applications. For each modality, we discuss how they modulate pain signaling across peripheral, spinal, and supraspinal levels. We also highlight limitations, challenges, and future directions for optimizing the use of NINPT. We aim to build a bridge between basic research on mechanisms of NINPT and their clinical implementation for chronic pain relief.

## Theoretical backgrounds of chronic pain

While acute and chronic pain both involve the pain pathway from nociceptors to brain, chronic pain arises from dysregulation of the balanced mechanisms that adaptively modulate pain signaling in the acute state. Elucidating the neurophysiological processes underlying this maladaptive transition across molecular, cellular, and systems levels will inform targeted interventions.

### Anatomy and physiology of pain pathways

**Fig. 1:**
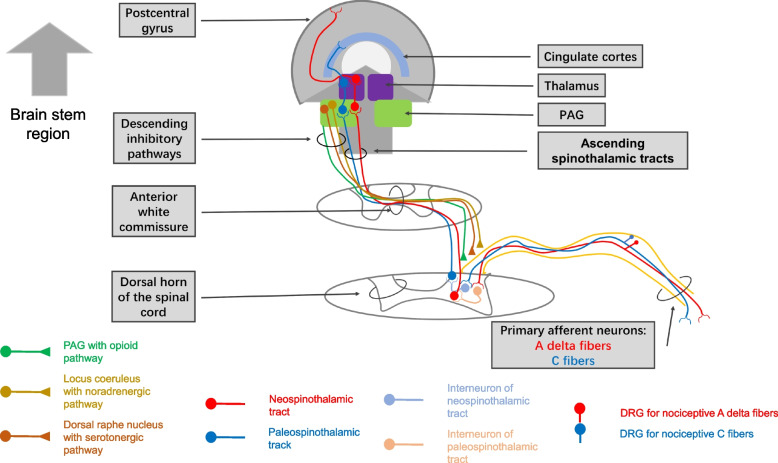
Ascending pain pathway from periphery to central. Note: PAG = periaqueductal gray

The pain pathway consists of three core components: nociceptors that detect pain signals, the spinal cord that transmits and modulates the signals, and the brain that processes and regulates the pain experience [26]. Nociceptors are specialized sensory receptors that respond to harmful stimuli by converting them into electrical signals. In chronic pain, nociceptors become hypersensitive and can be activated by normal sensory stimulation. These signals are transmitted by Aδ and C fibers to the dorsal horn of the spinal cord, where they synapse with second-order neurons [27] (Fig. 1).

The dorsal horn of the spinal cord is the first site where pain signals enter the spinal cord and undergo initial processing and modulation [26]. In chronic pain, the dorsal horn shows increased neuronal excitability and reduced inhibition, amplifying and dysregulating pain signal transmission. Neurons and neurotransmitters in the dorsal horn, like glutamatergic and GABAergic neurons, facilitate and inhibit pain signal conduction. Descending facilitation from the brainstem also enhances the dorsal horn’s role in augmenting pain signaling in chronic pain [27].

The second-order neurons then cross the midline or remain ipsilateral and ascend to the brain through tracts including the spinothalamic, spinoreticular, and spinomesencephalic tracts [26] (Fig. 1). In chronic pain, changes in the volume and connectivity of these tracts correlate with clinical pain intensity and modulation. Pain signals are processed and modulated in various brain regions, including the thalamus, brainstem, and cortex. Multiple brain areas exhibit functional and anatomical changes in chronic pain that disrupt normal pain modulation [28, 29].

**Fig. 2:**
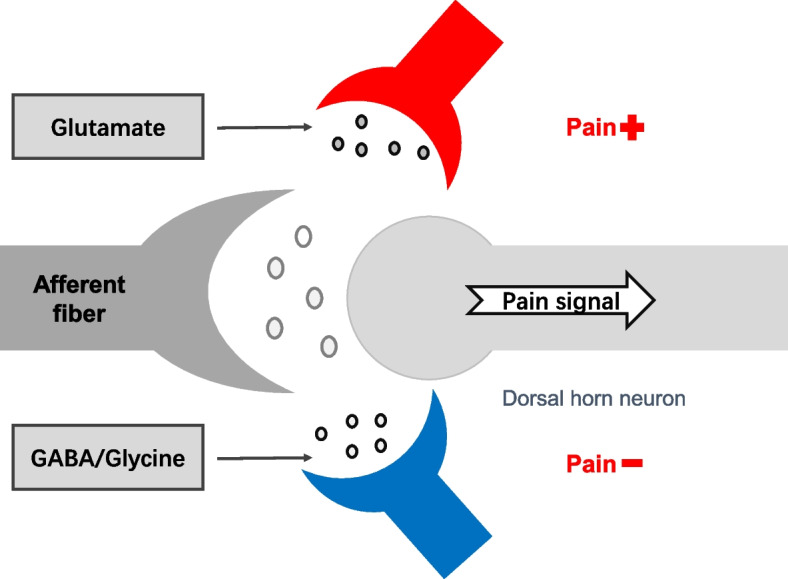
Modulation of pain signals in the spinal dorsal horn

Recent research has advanced our understanding of the mechanisms underlying pain signaling and modulation across the pain pathway. Targeting these maladaptive changes may help restore normal processing, facilitate adaptation and improve quality of life. At the nociceptor level, factors like cytokines, growth factors, and nerve injury regulate ion channels including sodium, potassium, calcium, and transient receptor potential channels [30, 31]. Neurotransmitter interactions in the spinal cord, such as glutamate, GABA, glycine, and substance P, play an essential role in synaptic transmission and plasticity of pain signals [32, 33] (Fig. 2).

**Fig. 3:**
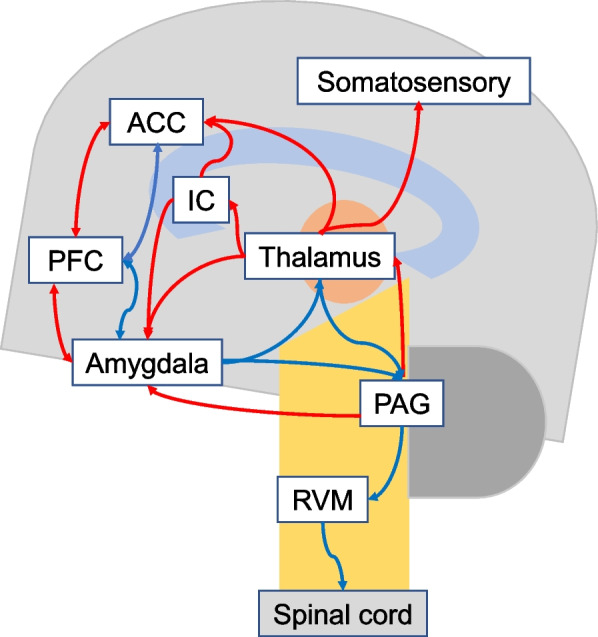
Interactions between pain modulatory brain regions. Note: The red lines represent the ascending nociceptive pathways that transmit pain signals, while the blue lines represent the descending anti-nociceptive pathways that modulate pain. PFC = prefrontal cortex; ACC = anterior cingulate cortex; IC = insula cortex; PAG = periaqueductal gray; RVM = rostral ventromedial medulla

Various brain regions, including the anterior cingulate cortex, insula, amygdala, prefrontal cortex, and periaqueductal gray, detect, process, and modulate pain signals by activating or inhibiting circuits involved in pain’s sensory, emotional, cognitive, and behavioral aspects [29, 34, 35]. The endogenous opioid system also regulates pain modulation through inhibiting signaling and enhancing analgesia. Changes in these modulatory systems underpin disrupted pain regulation in chronic pain (Fig. 3).

### Disruption of pain modulation

Pain modulation refers to the process by which nociceptive signals are regulated to achieve balanced sensitivity to potential threats. In acute pain, inhibition dominates over facilitation, keeping modulation intact. In chronic pain, modulation becomes impaired due to disrupted inhibition and enhanced facilitation across the pain pathway.

In the dorsal horn, normal modulation involves balanced excitatory and inhibitory controls by neurotransmitters like glutamate, GABA, and glycine [33]. Descending facilitation and inhibition from the brainstem also contribute to regulating pain sensitivity. In chronic pain, increased descending facilitation, impaired GABA/glycine inhibition, and neuroinflammation enhance dorsal horn neuron excitability, disrupting modulation. Prolonged nociceptive signaling leads to central sensitization, further amplifying pain signaling [36].

In the brain, the anterior cingulate cortex and periaqueductal gray are critical for modulation [37]. The anterior cingulate cortex exerts cognitive and emotional modulation, while periaqueductal gray connections provide downstream inhibitory control [38]. In chronic pain, reduced connectivity between these areas undermines enhanced pain inhibition. The prefrontal cortex also shows impaired cognitive modulation [29, 39]. Changes in endogenous modulatory systems, including opioids, further weaken inhibitory control.

Various interventions aim to rebalance modulation across the pain pathway. Targeting abnormal changes at all neuromatrix levels may help reinstate neural balance, regain function, and improve quality of life. A mechanism-based precision approach may address individual heterogeneity in chronic pain [40].

In summary, the progression from acute to chronic pain signifies a transition from an adaptable nociceptive system with modulated pain sensitivity to one dominated by unregulated signaling across the nervous system. Restoring modulation is key to limiting chronicity and optimizing outcomes. Overall, chronic pain arises from disruption of balanced mechanisms that regulate nociceptive signal perception and transmission in the normal acute pain state.

### Central sensitization: a driver of chronic pain

Central sensitization refers to the maladaptive upregulation of the central nervous system’s response to painful stimuli and normal sensory signals. Through descending facilitation, widespread hyperexcitability, impaired inhibition, and inflammatory processes, central sensitization manifests as an amplification of neural signaling along the neuraxis that drives chronic pain [41, 42]. It represents a key intermediate mechanism between acute and chronic pain. Neuroinflammation also plays an important role in the development and maintenance of central sensitization.

At the spinal level, constant nociceptive input from peripheral pain generators drives hyperexcitability of dorsal horn neurons, known as heterosynaptic facilitation [43]. Glutamate and inflammatory mediators activate NMDA receptors on dorsal horn neurons, initiating events that enhance neuronal excitability and synaptic plasticity [44]. Loss of inhibitory control mediated by neurotransmitters like GABA and glycine further amplifies signaling to higher brain centers. Descending facilitation from the brainstem exacerbates spinal hyperexcitability [44].

In the brain, key structures involved in the sensory, emotional and cognitive aspects of pain show altered activity, connectivity, and neurochemistry. For example, the anterior cingulate cortex exhibits elevated activity but reduced connectivity with the periaqueductal gray, diminishing engagement of descending inhibitory controls [45, 46]. The prefrontal cortex shows impaired cognitive control over pain [39]. Changes in the endogenous opioid system and microglial activation highlight the role of disrupted top-down modulation and neuroinflammation in perpetuating central sensitization.

Processes like long-term potentiation, epigenetic changes, and neuroimmune dysregulation have been implicated in the cellular and molecular processes underlying central sensitization. Targeting the mechanisms driving and maintaining central sensitization may help restore normal pain regulation, limit chronicity, and improve wellbeing. Treatment needs a systematic approach addressing changes across the pain neuromatrix and balancing neural modulation. Central sensitization represents a seminal mechanism in pain chronification demanding mechanism-based management [47]. Overall, central sensitization signifies an enduring state of maladaptive neuroplasticity across the neuraxis that amplifies pain transmission and disables endogenous modulation.

In summary, central sensitization promotes chronic pain by inducing a self-perpetuating cycle of enhanced signaling and diminished modulation across the pain neuraxis.

## Types of NINPT

NINPT encompass heterogeneous physical, psychological, and complementary therapies as well as other techniques that modulate multidimensional aspects of the pain pathway without invasive procedures. Understanding their differential effects and mechanisms of action at peripheral, spinal, and supraspinal sites will enable optimal translation and integration of these modalities to relieve chronic pain.

### Physical modalities

Physical modalities are interventions that use physical stimuli, such as electricity, heat, cold, or pressure, to modulate pain signals. Physical modalities can act on the peripheral, spinal, or supraspinal levels of the pain pathway, depending on the type, intensity, duration, and location of the stimuli. Some of the most common physical modalities are:

#### TENS

TENS is a method for relieving pain through the application of low-intensity electrical stimulation. However, repeated TENS applications may cause analgesic tolerance [48]. This technique is delivered via TENS devices which provide electrical stimulation through leads attached to adhesive electrodes. TENS devices include compact, battery-powered portable units as well as larger stationary models [49, 50].

At the peripheral level, TENS activates large myelinated Aβ fibers which inhibit nociceptive transmission in small Aδ and C fibers according to the gate control theory. This relies on presynaptic inhibition of nociceptive input at the dorsal horn [51, 52]. Molecular studies show TENS modulates the expression and activity of key pain-related ion channels on peripheral nerves, including voltage-gated sodium channels, TRP channels, inhibiting excitability and neurotransmission in nociceptor [53, 54].

In the spinal cord, functional magnetic resonance imaging (MRI) indicates TENS suppresses activity in Rexed laminae I-V of the dorsal horn, which transmit and modulate nociceptive signaling [55, 56]. Immunohistochemistry studies demonstrate TENS increases spinal expression of μ-opioid, δ-opioid, and cannabinoid receptors [57]. Electrophysiology studies show TENS reduces wind-up and potentiation of dorsal horn neurons by modulating NMDA receptors [58]. Repeated use may cause tolerance due to NMDA receptor desensitization [48].

In the brain, TENS alters activity and functional connectivity in regions including the thalamus, somatosensory cortex, anterior cingulate cortex, insula, prefrontal cortex, and periaqueductal gray, suggesting effects on sensory, cognitive, and affective dimensions of pain. Functional connectivity analysis reveals TENS strengthens top-down connectivity from the periaqueductal gray and prefrontal cortex to the anterior cingulate cortex [59]. Electroencephalogram (EEG) shows TENS increases alpha and beta oscillations that correlate with analgesia [60]. Molecular studies indicate TENS increases endogenous opioid and serotonin levels in brain regions involved in descending modulation [61].

Evidence shows TENS can reduce pro-inflammatory cytokines and modulate neuroimmune interactions involved in chronic pain [62]. It may also promote neural plasticity by stimulating neurotrophic factors like brain-derived neurotrophic factor (BDNF) and beta-endorphins [63].

Recent clinical trials support the efficacy of TENS for relieving neuropathic pain in conditions such as diabetic neuropathy, post-herpetic neuralgia, and neuropathic pain after spinal cord injury [64–66] (Additional file 1: Fig. S1). Randomized controlled trials demonstrate TENS reduces neuropathic pain intensity and improves physical function compared to sham TENS. The effects may involve altering peripheral nerve excitability and central sensitization [66, 67].

For osteoarthritis pain, meta-analyses of numerous randomized controlled trials indicate TENS applied to the knee joint decreases pain and improves physical function [68, 69]. Proposed mechanisms include reducing inflammation, activating descending inhibition, and stimulating analgesic neurotransmitter release. However, the evidence for TENS in relieving stiffness in osteoarthritis is inconsistent [69, 70]. Although some studies suggest TENS may reduce stiffness in osteoarthritis patients [69], the efficacy of TENS on osteoarthritis needs further verification.

In fibromyalgia, TENS applied to tender points provides analgesia and improves pain threshold compared to sham treatment [71]. Suggested mechanisms involve modulating substance P levels, increasing blood flow, and restoring abnormal pain processing.

While research has focused on the effects of TENS for osteoarthritis and fibromyalgia, future studies should evaluate its therapeutic potential for other common causes of chronic pain such as diabetic neuropathy.

TENS may have synergistic benefits when combined with pharmacological, psychological, physical, or complementary interventions as part of a multimodal approach. For example, TENS plus opioid therapy could improve analgesia while reducing opioid-related risks [72]. TENS combined with CBT may provide sensory-cognitive modulation of chronic pain [73]. Future research should explore optimal multimodal protocols incorporating TENS for diverse chronic pain conditions.

While TENS shows promise, larger, high-quality randomized controlled trials are needed to establish optimal protocols and long-term effectiveness for chronic pain relief. Emerging research on computational modeling and artificial intelligence provides new opportunities to optimize TENS stimulus parameters such as intensity, frequency, and pattern [74]. Advanced algorithms and neural networks could analyze individual patient factors to determine the optimal dosing strategy. Real-time closed-loop TENS systems with biofeedback may also enhance outcomes [75].

#### Heat therapy and cryotherapy

Heat therapy and cryotherapy refer to the application of thermal agents to tissue for pain relief. Heat therapy utilizes sources such as hot packs, paraffin wax, and infrared radiation to apply superficial or deep heating [76]. Cryotherapy employs cold agents such as ice packs, cold compresses, and refrigerant sprays to lower tissue temperature [76].

Heat therapy enhances blood flow and relaxes muscles, while activating thermosensitive TRP channels that modulate peripheral and central thermal sensitivity [77]. Neuroimaging studies show heat may activate endogenous opioid and cortical serotonergic systems involved in analgesia by improving connectivity between the thalamus, anterior cingulate cortex, and periaqueductal gray [76, 78]. Recent evidence suggests heat therapy reduces central sensitization markers including neuroinflammation and NMDA receptor activation [79, 80].

In contrast, cryotherapy limits inflammation and nerve conduction velocity through vasoconstriction and by suppressing release of neurotransmitters including serotonin and glutamate. This inhibits peripheral sensitization and nociceptive signals transmission [81]. Cryotherapy may also mitigate central sensitization by reducing membrane excitability, presynaptic transmitter release, and signal propagation in pain pathways. EEG studies reveal cryotherapy enhances alpha and beta wave rhythms, which may impact cognitive and attentional pain modulation [82, 83].

Emerging studies propose that heat and cold may also influence neural plasticity and central sensitization in chronic pain. Heat therapy appears to enhance neuroplasticity markers like BDNF and reduces central sensitization markers like NMDA receptor expression [79]. In contrast, cryotherapy may limit central sensitization by reducing neurotransmitter release, metabolism, and nerve conduction velocities [81].

Recent clinical trials demonstrate heat therapy reduces neuropathic pain in conditions like diabetic neuropathy, chemotherapy-induced peripheral neuropathy, and neuropathic pain after spinal cord injury [84–86] (Additional file 1: Fig. S1). The effects may involve modifying thermal hyperalgesia and central sensitization. Meta-analyses also indicate cryotherapy decreases neuropathic and nerve injury pain.

For osteoarthritis pain, heat and cold therapies applied to the affected joints provide analgesia and improve physical function versus placebo [87, 88]. The mechanisms may involve altering inflammation, muscle relaxation, and joint stiffness.

In fibromyalgia, localized heat and cryotherapy on tender points alleviates widespread pain and sensitivity compared to control conditions [89]. Possible effects include releasing muscle trigger points and restoring descending modulation.

In addition to osteoarthritis and fibromyalgia, future research should assess the effects of thermal therapies on other frequent causes of chronic pain like nerve injury and central sensitization.

Thermal therapies as adjuvants to pharmacologic treatment like NSAIDs, opioids, and membrane stabilizers demonstrate enhanced analgesia in chronic postoperative pain and neuropathic pain [90, 91]. Heat and cold may exert synergistic effects through sensory-discriminative pathways when combined with analgesics.

Integrating thermal therapies into exercise and physical therapy improves outcomes for chronic musculoskeletal pain compared to either treatment alone. The combination optimizes physiological and psychosocial factors influencing chronic pain [92, 93].

While thermal therapies demonstrate short-term analgesia, long-term effectiveness and optimal protocols require further research through high-quality, large-scale randomized controlled trials with extended follow-up periods [94]. There is a lack of evidence on the duration of pain relief and ideal treatment frequency and dosing [92]. Future studies should investigate long-term impacts on pain reduction, functional improvement, side effects, and quality of life.

Individualization of thermal therapies is essential to maximize analgesic effects while ensuring safety and tolerability. Treatment plans should be tailored based on pain location and type, patient preferences, and degree of desired temperature change [92, 95]. Factors like comorbidities, age, sex, and individual variation in thermal sensitivity and endogenous analgesia systems may influence outcomes. Real-time monitoring and feedback could help determine optimal personalized protocols.

Proper safety precautions should be taken during delivery of heat and cold to avoid adverse events like burns, frostbite, or nerve damage. Heat therapy risks include dehydration and aggravated inflammation if overheated [96]. Prolonged exposure to extreme cold may cause irreversible nerve injury [94]. Application guidelines regarding duration, temperature thresholds, and monitoring should be followed, especially in special populations. Overall, individualization of thermal therapies while ensuring safety is key to optimizing effectiveness.

#### Massage therapy

Massage therapy has emerged as an effective method for managing chronic pain with promising results in clinical trials [12]. However, the underlying mechanisms by which massage relieves chronic pain remain unclear.

The gate control theory suggests massage activates spinal inhibitory interneurons to suppress pain signal transmission to the central nervous system. Massage may activate cutaneous mechanoreceptors and modulate spinal processing of tactile inputs [97]. Neuroimaging studies reveal massage increases release of endogenous opioids like beta-endorphins, enhancing descending modulation from the periaqueductal gray [98]. Massage regulates autonomic balance by increasing parasympathetic activity and reducing inflammatory cytokines [12].

Massage could modulate the hypothalamic–pituitary–adrenal axis and sympathetic nervous system involved in regulating inflammation and stress responses. This may decrease pro-inflammatory cytokines like Interleukin (IL)-6 and tumor necrosis factor alpha (TNF-α) while increasing anti-inflammatory cytokines like IL-10 [12]. Downregulation of pro-inflammatory cytokines may reduce sensitization of peripheral nociceptors and central pain pathways.

Recent evidence indicates massage therapy could prevent or reverse central sensitization by normalizing aberrant sensory input, improving circulation, decreasing trigger points and muscle tension, and optimizing descending inhibition. Brain stimulation studies suggest massage normalizes functional connectivity in chronic pain-related neural networks including the default mode, salience and sensorimotor systems [99].

Recent randomized controlled trials demonstrate massage therapy decreases neuropathic pain in diabetic neuropathy, chemotherapy-induced peripheral neuropathy, and nerve injury conditions [100–102] (Additional file 1: Fig. S1). The effects may involve modulating neural hypersensitivity and plasticity.

For osteoarthritis pain, massage applied to affected joints consistently reduces pain and improves physical function compared to control interventions. Massage alleviates pain by enhancing joint mobility, muscle relaxation, and circulation [103].

In migraine and tension headaches, massage therapy targeting neck, head, and facial muscles significantly reduces headache frequency, intensity, and disability versus placebo [104]. The mechanisms likely involve inhibiting peripheral and central sensitization.

As an adjuvant, massage combined with pharmacotherapy like NSAIDs, tricyclics, and anticonvulsants shows greater analgesia than drugs alone in chronic back pain and fibromyalgia [105, 106]. Massage may enhance drug absorption and act synergistically on pain pathways.

For musculoskeletal conditions like osteoarthritis, neck pain, and shoulder pain, massage integrated into exercise and physical therapy improves outcomes including pain relief, function, and quality of life compared to either treatment alone [12, 107].

While massage demonstrates short-term pain relief, evaluating its long-term efficacy requires assessing multidimensional outcomes including sustained pain reduction, functional improvement in daily activities, quality of life enhancement, patient adherence, and side effects [108]. Future studies should utilize standardized measures over extended periods to determine optimal treatment duration, frequency, and techniques for maintaining benefits [109].

Advances in genomics and neuroimaging could provide biomarkers to guide individualized massage therapy based on a patient’s genetic profile, endogenous opioid function, and baseline neural activity in pain processing regions. This precision approach could identify optimal massage methods and dosing according to the underlying pain mechanisms for each patient [110].

Special considerations should be given to applying massage in vulnerable populations like the elderly, pregnant women, and patients with cancer or recent trauma/fractures [101, 111]. Precautions are needed regarding pressure, positioning, potential drug interactions, and contraindications. Milder massage techniques with supportive aids may be required to ensure safety and tolerability. Overall, an individualized approach factoring patient characteristics is key to maximize massage therapy benefits.

#### Ultrasound therapy

Ultrasound therapy utilizes high-frequency sound waves to promote tissue healing and relieve chronic pain [112]. It alleviates chronic pain by activating descending inhibitory pathways, modulating neurotransmitters and neuropeptides, reducing inflammation and central sensitization, and stimulating tissue regeneration [113].

In the spinal cord, animal studies suggest ultrasound may inhibit dorsal horn neuron excitability and suppress release of nociceptive neurotransmitters including glutamate, substance P and CGRP, reducing pain signal transmission to the brain [114, 115]. Ultrasound also enhances spinal release of serotonin, norepinephrine, and endogenous opioids that activate descending inhibitory pathways.

Ultrasound may activate cutaneous thermosensitive TRP ion channels to alter peripheral and central pain sensitivity thresholds [116]. Emerging evidence suggests ultrasound increases BDNF, promoting synaptic plasticity and analgesia in pain pathways [117]. Ultrasound therapy may also restore balanced levels of pro- and anti-inflammatory cytokines to mitigate peripheral and central sensitization. It could increase μ-opioid receptor density and decrease inflammatory mediators like cyclooxygenase-2 (COX-2) to reduce nociceptor sensitization [118, 119].

Ultrasound may influence neural plasticity by stimulating neurogenesis, angiogenesis, and synaptogenesis in pain modulatory regions including the periaqueductal gray, hippocampus, and prefrontal cortex [120, 121]. It can promote tissue repair through enhancing cell proliferation, collagen regeneration, and blood flow. Recent studies propose ultrasound may mitigate central sensitization and chronic pain by reducing NMDA receptor activation, neuron excitability, neuroinflammation, and oxidative stress in the spinal cord, thalamus, and pain modulatory areas [122, 123]. Neuroimaging shows ultrasound therapy optimizes functional connectivity in chronic pain-related neural networks [124].

Recent clinical trials demonstrate therapeutic benefits of ultrasound for neuropathic pain conditions like diabetic neuropathy, post-herpetic neuralgia, nerve injury pain, and neck pain [124–127] (Additional file 1: Fig. S1). The effects may involve modulating nerve excitability, neuroinflammation, and neural plasticity.

For fibromyalgia, ultrasound therapy applied to tender points alleviates widespread pain and sensitivity compared to sham treatment, potentially by stimulating trigger point release and enhancing descending inhibition [128].

As an adjuvant therapy to NSAIDs, membrane stabilizers and opioids, ultrasound demonstrates enhanced analgesia compared to medication alone for chronic back pain, osteoarthritis, and neuropathic pain, possibly through additive sensory-discriminative effects [129–131].

Integrating ultrasound into exercise and physiotherapy improves outcomes including pain relief and function versus individual treatments alone for chronic neck, shoulder, and knee pain, optimizing multiple factors influencing chronic pain [128, 132, 133].

While ultrasound therapy demonstrates short-term pain relief, studies evaluating its long-term efficacy and optimal treatment regimens are lacking [134]. There is limited evidence on sustained analgesic effects, ideal dosing frequency and duration, number of sessions required, and prevention of symptom recurrence. Further research through high-quality randomized controlled trials with extended follow-up is warranted to establish protocols maximizing long-term outcomes.

Individual patient factors like age, sex, comorbidities, genetics, baseline pain sensitivity, and endogenous opioid function may influence ultrasound treatment response [113]. Identification of biomarkers predicting treatment outcomes could enable personalized protocols tailored to underlying pain mechanisms. Advances in genomics, neuroimaging, and machine learning techniques could facilitate individualized ultrasound therapy [135].

Potential safety concerns with ultrasound include tissue overheating and cavitation effects. Guidelines on intensity, frequency, duration of application, and monitoring techniques should be followed to avoid tissue damage. Using pulsed ultrasound and keeping transducer movement constant during treatment could improve safety [119]. Overall, individualization of ultrasound therapy while ensuring appropriate treatment parameters is key to optimizing its clinical application.

#### Light therapy

Light therapy utilizes specific wavelengths of light, such as far infrared and laser, to promote tissue healing and relieve chronic pain [134, 136]. Light therapy demonstrates analgesic effects when applied transcranially to modulate cortical activity as well as when applied locally to the skin surface above painful areas. Light therapy may achieve analgesic effects through mechanisms involving retinal photoreceptors, inflammation, mitochondrial function, and neural activity.

Infrared light may increase nitric oxide and stimulate mitochondrial function, while laser therapy may activate neural pathways mediating analgesia [137, 138]. Light activates retinal opsins and photosensitive proteins that influence neurotransmitters and activity in pain processing brain regions. Light therapy increases BDNF, promoting synaptic plasticity and analgesia. It regulates oxidative stress and inflammation through effects on reactive oxygen species and cytokines [139].

Recent studies propose light therapy may mitigate central sensitization and chronic pain by reducing NMDA receptor activation and neuron excitability in pain pathways [140]. Light also improves functional connectivity between chronic pain-related brain regions including thalamus, somatosensory cortex, insula, cingulate cortex, prefrontal cortex, and periaqueductal gray [139].

Light therapy demonstrates analgesic effects when applied transcranially to modulate cortical activity as well as when applied locally to the skin surface above painful areas [141, 142]. In clinical studies, light therapy demonstrates analgesic effects in diverse chronic pain conditions beyond osteoarthritis, back and neck pain [143, 144]. Recent clinical trials show pain relief with light therapy in fibromyalgia, diabetic neuropathy, chemotherapy-induced peripheral neuropathy, phantom limb pain, post-herpetic neuralgia, and complex regional pain syndrome [145–150] (Additional file 1: Fig. S1). The effects may involve modulating peripheral and central neural sensitivity.

As an adjunctive treatment, light therapy combined with pharmacological agents like NSAIDs and opioids shows enhanced analgesia compared to medication alone in randomized trials for low back pain, arthritis pain, and neuropathic pain [151, 152]. Light therapy could exert additive effects through sensory-discriminative pathways.

Integrating light therapy into exercise, physical therapy, and manual therapy programs results in improved outcomes including pain relief and functional gains versus individual treatments alone for chronic musculoskeletal pain disorders. The combination optimizes multiple physiological and neuromodulatory factors influencing chronic pain [134, 153].

In pediatric populations, light therapy reduces pain and discomfort associated with needle-related procedures such as venipuncture and intravenous cannulation based on clinical studies [154]. The mechanisms may involve local vascular and neural effects.

Proper safety precautions should be taken during delivery of light therapy to avoid adverse effects like burns and phototoxicity. Guidelines on intensity, frequency, duration of application, and monitoring techniques should be followed to prevent tissue damage, especially in vulnerable populations [155]. Using intermittent light delivery and monitoring skin temperature could improve safety. Overall, individualization of light therapy while ensuring appropriate treatment parameters is key to optimizing its clinical utility.

### Psychological interventions

Psychological interventions are interventions that use cognitive, emotional, or behavioral strategies to modulate pain signals. Psychological interventions can effectively modulate chronic pain through influencing supraspinal pain processing. By altering activity and connectivity in brain regions involved in perception, emotion, cognition, and behavior, such as the thalamus, somatosensory cortex, insula, anterior cingulate cortex, amygdala, prefrontal cortex, and hippocampus, psychological techniques may relieve suffering and restore function.

#### CBT

CBT is a psychotherapy that aims to change maladaptive thoughts and behaviors related to chronic pain. It works by changing the way people perceive their pain, by challenging negative thoughts and enhancing positive ones through cognitive restructuring and cognitive reappraisal [13]. CBT also targets emotional and behavioral responses to chronic pain, such as fear, anxiety, depression, and anger, by using techniques like exposure therapy, relaxation training, behavioral activation, and emotion regulation [13, 156]. Additionally, CBT addresses lifestyle factors that can impact pain, such as sleep hygiene, diet, physical activity, and social support [156].

Research shows CBT increases endogenous opioid β-endorphin expression and reduces inflammatory factors like IL-6 and TNF-α, suggesting potential analgesic and anti-inflammatory mechanisms [157].

CBT may modulate activity and connectivity of brain regions involved in pain processing, including the prefrontal cortex, anterior cingulate cortex, insula, and amygdala [158]. It regulates pain through “gating” mechanisms, engaging descending modulatory pathways releasing neurotransmitters like opioids, serotonin, and norepinephrine to inhibit spinal nociceptive signaling.

Additionally, CBT improves coping, relaxation, and emotion regulation through techniques like exposure therapy and behavioral activation, targeting pain-related emotions like fear, anxiety, depression, and anger [156].

In addition to low back pain, fibromyalgia, migraine, and neuropathic pain, CBT demonstrates clinical utility across an extensive range of chronic pain disorders [159–161]. Recent studies reveal CBT reduces pain and improves function in chronic neck pain, rheumatoid arthritis, irritable bowel syndrome, temporomandibular disorders, chronic abdominal pain, and chronic pelvic pain [9, 162–166] (Additional file 1: Fig. S2). The effects involve altering pain perception, catastrophizing, and coping strategies. For cancer-related pain, CBT combined with pharmacological therapy more effectively reduces pain compared to drug therapy alone, possibly by enhancing pain coping and medication adherence [167].

As an adjuvant treatment, CBT integrated with exercise therapy, massage therapy, and chiropractic care shows greater improvements in pain and function than individual therapies alone for chronic low back pain and osteoarthritis [168]. CBT may have synergistic effects by optimizing sensory, cognitive, and emotional dimensions.

While CBT demonstrates efficacy for chronic pain, long-term benefits and optimal treatment protocols need further research through high-quality randomized controlled trials and longitudinal studies [169]. There is limited evidence on the durability of CBT-induced pain relief and functional gains after treatment cessation. Studies should evaluate sustained effects, ideal session frequency/duration, and value of booster sessions.

Emerging techniques like neuroimaging, EEG, and machine learning may identify biomarkers to guide individualized CBT based on patient characteristics predicting treatment response. For example, functional MRI could indicate baseline neural signatures forecasting CBT outcomes. VR could also be integrated to enhance personalization of CBT content and delivery [170, 171].

CBT is considered very safe with minimal risks. However, comorbid psychological or cognitive deficits may require pre-screening and adapted protocols under supervision to prevent adverse events like anxiety, confusion, or withdrawal [172]. Supportive aids and graded exposure may improve safety and tolerability. Overall CBT demonstrates an excellent safety profile but techniques should be tailored for vulnerable populations.

#### Hypnosis

Hypnosis is a psychotherapeutic technique that utilizes suggestions to induce a state of altered consciousness, characterized by focused attention, relaxation, and heightened responsiveness to suggestions. This technique operates by altering the perception of pain through modifying the sensory, affective, and cognitive components of chronic pain [173]. Hypnosis also works by modifying the emotional and behavioral responses to chronic pain, by reducing fear, anxiety, depression, and anger, and increasing relaxation, coping, and self-efficacy [174].

Recent neuroimaging studies using functional MRI, positron emission tomography (PET), and EEG reveal hypnosis modulates activity and functional connectivity in multiple brain regions involved in pain perception and modulation, including the anterior cingulate cortex, prefrontal cortex, insula, thalamus, amygdala, and periaqueductal gray [175, 176]. Hypnosis may achieve analgesia by enhancing top-down regulation from cortical and subcortical regions to attenuate ascending nociceptive signaling.

On the molecular level, hypnosis has been found to alter levels of neurotransmitters and neuropeptides involved in pain modulation, including endogenous opioids, serotonin, dopamine, and cholecystokinin [177]. Hypnosis may engage descending inhibitory mechanisms mediated by opioids and monoamines to suppress spinal nociceptive transmission [178].

Recent metabolomics studies indicate hypnosis can regulate various metabolic pathways involved in inflammation, oxidative stress, and energy metabolism that may underlie its analgesic effects. Hypnosis normalizes levels of metabolites related to prostaglandin/leukotriene synthesis, nitric oxide production, and nucleotide metabolism [179].

Emerging epigenetics research indicates hypnosis may exert long-term effects on pain perception by altering chromatin structure and gene expression patterns. Hypnosis induces chromatin remodeling at the promoters of genes encoding pain modulatory factors like opioid receptors, BDNF, and catechol-o-methyltransferase (COMT). This leads to lasting changes in the transcription of these analgesic mediators [180].

Hypnosis demonstrates clinical utility across diverse chronic pain conditions including low back pain, arthritis, fibromyalgia, migraine, cancer pain, and more [181–185] (Additional file 1: Fig. S2). The analgesic effects involve modulating sensory, cognitive, and emotional dimensions of pain.

Importantly, recent evidence highlights the value of hypnosis as an adjunct to optimize pharmacological, physical, and psychological therapies through mechanisms like enhancing descending inhibition, altering pain perception, optimizing attentional processes, and reinforcing positive expectancies and placebo effects [181].

Hypnosis combined with NSAIDs, opioids, and anesthetics demonstrates enhanced analgesia and reduced medication side effects in both acute and chronic pain settings. It improves outcomes of invasive procedures like surgery and childbirth [186]. When integrated with physiotherapy, massage, acupuncture, chiropractic care, and exercise therapy, hypnosis leads to greater improvements in pain, function, and quality of life than individual therapies alone for various musculoskeletal and neuropathic disorders [187].

Hypnosis as an adjuvant to chemotherapy alleviates cancer-related pain and discomfort more effectively than chemotherapy alone [167]. Hypnosis combined with VR therapy demonstrates enhanced analgesia for pediatric acute and procedural pain management [188].

Realizing the full potential of hypnosis will require advances in personalized, precision protocols. Techniques like neuroimaging, EEG, genomics, and machine learning may identify biomarkers to guide individualized hypnosis therapy based on a patient’s genetics, psychology, brain patterns, and outcomes [189]. This could help optimize tailored hypnosis delivery and suggestions for each patient’s needs and pain mechanisms.

Further research should also explore optimal combination strategies for hypnosis as part of multidisciplinary pain management. In particular, studies investigating synergistic effects of hypnosis with newer modalities like VR, brain stimulation, and biologics are warranted. Large collaborative trials evaluating multi-component protocols that integrate hypnosis with pharmacological, psychological and physical therapies will help advance integrative care models.

#### Mindfulness-based interventions

Mindfulness-based interventions involve using mindful awareness of the present moment without judgment to address pain [190, 191]. These techniques influence the cognitive, affective, and sensory aspects of pain processing.

Neuroimaging studies reveal mindfulness modulates activity and connectivity of key pain-related brain regions like the prefrontal cortex, anterior cingulate cortex, insula, thalamus, and amygdala [192]. Mindfulness may engage top-down regulation from prefrontal cortex and anterior cingulate cortex to inhibit maladaptive pain signaling.

Accumulating evidence indicates mindfulness impacts pain modulatory neurotransmitters including endogenous opioids, endocannabinoids, serotonin, and dopamine [193]. Genomics and proteomics studies indicate mindfulness reduces expression of inflammatory genes and proteins, mitigating peripheral and central sensitization [194].

Mindfulness regulates the autonomic nervous system, restoring sympathetic-parasympathetic balance and normalizing cardiovascular, hypothalamic–pituitary–adrenal axis, and immune system functions involved in chronic stress and pain chronification [195].

Importantly, mindfulness normalizes aberrant connectivity within and between default mode, salience, central executive, and sensorimotor brain networks that underlie cognitive, affective, and sensory processing of pain [196]. This systems-level mechanism provides a neural circuitry framework for mindfulness-based analgesia.

Mindfulness-based interventions demonstrate clinical efficacy across extensive chronic pain disorders beyond fibromyalgia and back pain, including arthritis, headaches, diabetic neuropathy, cancer pain, postoperative pain, irritable bowel syndrome, chronic pelvic pain, temporomandibular joint disorder, and more [197–205] (Additional file 1: Fig. S2). The analgesic effects involve modulating cognitive, sensory, and emotional aspects of the multidimensional pain experience.

Notably, mindfulness plus morphine demonstrates greater analgesic effects and fewer side effects than morphine alone for cancer pain, as mindfulness enhances pain coping and expectancies [201]. When incorporated into physiotherapy and massage therapy, mindfulness leads to significantly greater improvements in chronic low back pain than individual therapies alone by optimizing attentional processes [9]. This approach combined with acceptance commitment therapy is more efficacious for reducing arthritis pain and improving mobility than either alone, as the combination reinforces adaptive coping. Additionally, mindfulness plus CBT provides superior migraine symptom relief and quality of life versus either therapy alone by targeting sensory, cognitive and emotional aspects [206].

While promising, there are limitations of mindfulness-based interventions that warrant consideration. Optimal mindfulness protocols tailored to different chronic pain populations and delivery methods need further investigation through high-quality randomized controlled trials and mechanistic studies. Long-term durability of treatment effects, ideal “dosing,” and factors influencing individual variability in response remain unclear. Issues regarding accessibility and patient adherence must also be addressed [207].

Moving forward, several areas require attention to optimize mindfulness-based pain therapies. Development of adapted mindfulness protocols personalized for pediatric, geriatric, and cognitively impaired populations should occur through human-centered design approaches. Optimization of mindfulness training delivery methods, such as VR and smartphone apps, is needed to enhance engagement and accessibility [208, 209]. Further research on mindfulness combination strategies with emerging modalities like neurostimulation, psychedelics, and immunotherapy is essential to develop integrative pain care models.

Overall, continued investigation into mechanisms, personalized protocols, delivery methods, and combination strategies will facilitate unlocking the full potential of mindfulness-based interventions as part of integrative care paradigms for diverse chronic pain disorders.

#### Placebo effect

Placebo and nocebo effects represent neurophysiological responses that alter pain perception based on expectations and beliefs regarding treatment [210]. Optimizing placebo analgesia and mitigating nocebo hyperalgesia can enhance pain management outcomes. Elucidating the mechanisms of these effects informs strategies to amplify the benefits of pain treatments [210].

Neuroimaging studies using functional MRI reveal placebo and nocebo effects modulate activity and connectivity of key pain modulatory regions like periaqueductal gray, rostral anterior cingulate cortex, prefrontal cortex, amygdala, and hippocampus [211].

PET studies indicate placebo impacts release of endogenous opioid peptides involved in descending inhibition of nociception [212]. Genomics research shows placebo response relates to polymorphisms in genes of the endogenous opioid system [213].

Proteomics analyses demonstrate placebo treatment regulates expression of proteins related to inflammation, neuroplasticity, and oxidative stress. These molecular effects likely contribute to placebo analgesia [214].

Importantly, placebo and nocebo alter aberrant functional connectivity within key brain networks processing sensory, cognitive, and emotional dimensions of pain, including default mode, salience, and central executive networks [15, 211].

Beyond pain models and experimental settings, clinical studies demonstrate placebo treatments provide statistically and clinically meaningful pain relief in extensive chronic pain disorders including low back pain, osteoarthritis, neuropathic pain, fibromyalgia, irritable bowel syndrome, visceral pain, migraine, and tension headaches, among others [211, 215–221] (Additional file 1: Fig. S2).

Research shows placebo effects have clinical utility when combined with active pharmacological and non-pharmacological therapies for chronic pain. Placebo augmentation of opioid therapy results in greater analgesia at lower drug doses, enabling dose reduction [219]. Placebo combined with NSAIDs or anticonvulsants also enhances pain relief compared to the drugs alone [216]. Additionally, placebo integrated into physiotherapy, massage, acupuncture, mindfulness, and exercise therapy leads to significantly greater improvements in chronic pain versus individual therapies [222].

While promising, there are challenges and limitations in leveraging placebo effects that warrant consideration. The nature of placebo response variability between individuals and unpredictability over time require further elucidation. Ethical concerns regarding deception in placebo administration need addressing [215, 223].

Moving forward, optimizing clinical placebo applications to enhance pain management requires: Standardization of placebo procedures/controls in clinical trials to enable meaningful comparisons. Studies on ethical methods to harness placebo that minimize deception by enhancing positive patient-provider relationships, psychological factors and contextual cues [215]. Investigation of placebo in multimodal approaches combining pharmacotherapy, physical therapy, psychotherapy, and complementary medicine.

Overall, an integrative research framework elucidating the neurobiological, psychological, and ethical dimensions of placebo effects will enable their effective integration into individualized care models to optimize outcomes across diverse pain conditions.

### Complementary and alternative therapies

Complementary and alternative therapies are interventions that are not considered part of the conventional medical system, but are used to complement or replace it. Complementary and alternative therapies encompass various techniques that may relieve chronic pain through modulating physiological and psychological factors. Some of the promising options include acupuncture, yoga, tai chi, exercise, and music therapy.

#### Acupuncture

Acupuncture refers to stimulation of traditional Chinese acupoints. While acupuncture originally involved inserting needles into the body, this section focuses specifically on non-invasive techniques, such as laser or electro-stimulation, to stimulate acupoints. Acupuncture involves stimulating traditional acupoints along meridians to modulate pain signaling. Both manual and electrical stimulation may activate Aδ and C afferent fibers, triggering release of endorphins, enkephalins, and dynorphins that bind to opioid receptors in the periphery, spinal cord, and brain to inhibit nociception [224].

Acupuncture increases local release of adenosine, an endogenous anti-nociceptive agent, which inhibits pain transmission via adenosine A1 receptors based on biochemical assays [225]. Functional MRI reveals acupuncture modulates limbic areas involved in affective pain dimensions and activates anti-nociceptive regions like the periaqueductal gray [226].

Acupuncture elevates central levels of serotonin, norepinephrine, and dopamine, engaging descending inhibitory pathways according to microdialysis studies. Metabolomics analysis indicates acupuncture regulates glucose and lipid metabolism, purine metabolism, and arachidonic acid metabolism involved in anti-inflammatory effects [227, 228].

Emerging evidence suggests acupuncture may reverse central sensitization by reducing NMDA receptor activation, neuroinflammation, and oxidative stress in the spinal cord and pain modulatory brain regions based on immunohistochemistry and polymerase chain reaction (PCR) studies [229].

Clinical studies demonstrate the analgesic effects of acupuncture across diverse chronic pain conditions. Beyond low back pain and knee osteoarthritis, clinical trials show acupuncture analgesic effects in cancer-related pain, dysmenorrhea, migraine, tension headaches, abdominal pain, and chemotherapy-induced peripheral neuropathy, potentially by modulating sensory, affective, and cognitive pain dimensions [230–237] (Additional file 1: Fig. S3).

Importantly, research shows acupuncture enhances chronic pain relief when combined with pharmacological and non-pharmacological therapies. For example, acupuncture plus morphine demonstrates greater analgesic effects and fewer side effects than morphine alone for cancer pain [238]. Acupuncture integrated into physiotherapy programs yields superior improvement in mobility and function compared to either therapy alone for knee osteoarthritis [231]. This highlights the synergistic benefits of acupuncture through modulating multiple mechanisms when combined with other treatment modalities.

While acupuncture demonstrates potential for certain chronic pain conditions, there are limitations warranting consideration [239]. The mechanisms underlying acupuncture analgesia remain incompletely understood. Clinical efficacy varies for different pain disorders and individuals. Sham-controlled studies reveal a large placebo component in acupuncture effects. Safety issues like infection and pneumothorax should be avoided with proper technique and single-use needles.

Moving forward, optimization of acupuncture through computational modeling, neuroimaging, and machine learning could help identify biomarkers predicting response. Integration of electrical and non-invasive laser stimulation may enhance effects while avoiding adverse events. Evidence-based guidelines are needed to inform optimal acupuncture protocols tailored to each chronic pain condition and patient. High-quality randomized controlled trials with extended follow-up should evaluate long-term analgesic effects and impact on function, side effects, and quality of life. Development of acupuncture training programs should emphasize both traditional techniques and emerging evidence [239]. Overall, advancing acupuncture therapy through rigorous research will help unlock its potential within integrative care paradigms for chronic pain management.

#### Yoga and tai chi

Yoga and tai chi are mind–body practices that may relieve chronic pain and improve wellness through harmonizing physiological and psychological factors. Yoga and tai chi are ancient practices originating from India and China, respectively, which involve physical postures, movements, breathing techniques, and mental focus. They share with modern physical exercise the ability to balance key functions and cultivate optimal pain management [240, 241].

At the molecular level, studies indicate yoga and tai chi increase anti-inflammatory cytokines like IL-10 and IL-4, while reducing pro-inflammatory cytokines such as TNF-α, IL-6, and IL-1β [242, 243]. These effects help counteract chronic inflammatory processes underlying chronic pain conditions.

Functional MRI studies reveal yoga and tai chi can optimize abnormal functional connectivity between key pain-related brain regions. For instance, yoga normalizes altered connectivity between the default mode network and executive control network, which is associated with clinical pain severity [244]. Tai chi improves connectivity between the basal ganglia and sensorimotor cortex, which correlates with restored motor function [245]. Modulating dysfunctional connections in pain-related neural circuitry is crucial for translating neuroimaging findings into clinical utility.

PET imaging demonstrates yoga and tai chi increase release of endogenous opioids, serotonin, and dopamine in pain modulatory pathways including the periaqueductal gray, amygdala, and nucleus accumbens [246]. This activates descending inhibition of nociception.

Advanced EEG signal analysis indicates yoga and tai chi alter theta, alpha, and gamma oscillations associated with sensory, cognitive, and affective processing of pain [244]. This improves neural processing and integration of the pain experience.

Metabolomics studies show yoga and exercise induce changes in metabolites related to energy metabolism, oxidative stress, and inflammation. These biochemical effects may underlie their impact on pain [247, 248].

Epigenetic analyses reveal yoga and tai chi facilitate chromatin remodeling and alter miRNA expression patterns related to pain and analgesia [249]. These lasting changes may mediate long-term impact.

In addition to low back pain and osteoarthritis, emerging clinical evidence demonstrates yoga, tai chi, and physical exercise reduce pain and improve function in other chronic pain conditions, including fibromyalgia, migraine, neuropathic pain, cancer pain, multiple sclerosis pain, and inflammatory bowel disease pain [250–255] (Additional file 1: Fig. S3).

For example, several randomized controlled trials show yoga provides clinically meaningful improvements in pain, fatigue, and quality of life in fibromyalgia patients [256]. As an adjunctive therapy, yoga significantly reduces migraine headache frequency, intensity, and disability compared to pharmacotherapy alone [257].

Exercise training alleviates cancer-related pain and chemotherapy-induced peripheral neuropathy based on multiple studies [167]. Tai chi demonstrates significant analgesic effects in peripheral neuropathy patients, enabling reduced use of analgesics [258].

For instance, the combination of yoga and pregabalin is more effective than either therapy alone in reducing neuropathic pain and improving sleep based on a recent randomized controlled trial. Yoga integrated into physiotherapy programs results in superior outcomes compared to individual therapies for chronic low back pain [250]. Mindfulness meditation plus tai chi shows greater improvements in knee osteoarthritis pain and physical function versus either intervention alone [259].

While promising, there are limitations warranting consideration, including unclear optimal protocols for different chronic pain populations, factors influencing individual variability in treatment response, and need for further research on long-term analgesic durability through high-quality longitudinal studies. Accessibility and adherence barriers should also be addressed.

Moving forward, several areas need attention to optimize clinical applications of yoga, tai chi, and exercise for chronic pain. Development of adapted protocols personalized to patient characteristics and needs through predictive algorithms and machine learning is key. Optimization of therapy delivery methods, such as VR, telerehabilitation, and mobile apps, is essential to enhance engagement [260]. Combination strategies with emerging modalities like neurostimulation, nutraceuticals, and biologics should be explored. Large pragmatic trials on multi-component lifestyle programs integrating yoga, tai chi, and exercise are needed to inform integrative care models.

In summary, continued research on mechanisms, predictive biomarkers, delivery methods, and combination approaches will help realize the full potential of yoga, tai chi, and physical exercise as accessible, low-risk interventions that can be integrated into holistic paradigms for chronic pain management.

#### Music therapy

Music therapy, which employs music interventions to achieve therapeutic goals, encompasses a variety of techniques such as improvisational music therapy, song writing, music performance, and music listening. These techniques may be a promising and safe non-pharmacologic pain management option for patients with chronic pain [261, 262].

Neuroimaging shows music therapy modulates activity and connectivity in areas like thalamus, somatosensory cortex, cingulate cortex, insula, amygdala, prefrontal cortex, and nucleus accumbens [263]. Recent research proposes music therapy may normalize dysfunctional connectivity between default mode, salience, central executive, and reward networks that underlie sensory, affective, and cognitive dimensions of chronic pain [264]. It may reverse maladaptive neuroplasticity and limit central sensitization.

EEG, functional MRI, and computational modeling could help predict individual pain relief response and optimize music therapy protocols. Combining music with pharmacological, physical, or psychological pain treatments may have synergistic effects through peripheral and central mechanisms.

Recent clinical trials demonstrate music therapy decreases pain in additional chronic pain conditions including low back pain, arthritis, migraine headache, chronic neck, and shoulder pain [265–268] (Additional file 1: Fig. S3). Music alleviates both nociceptive and neuropathic pain through effects on sensory, cognitive, and emotional dimensions.

Integrating music therapy into physical therapy programs improves outcomes including pain relief, mobility, and quality of life versus physical therapy alone for osteoarthritis, rheumatoid arthritis, and chronic neck pain [269]. Music optimizes physiological and psychological influences on chronic pain.

Music therapy combined with CBT enhances pain coping and reduces disability compared to either therapy alone for patients with chronic widespread pain [270]. Music therapy optimizes multiple top-down mechanisms when combined with psychological therapies.

While music therapy demonstrates acute analgesic effects, studies evaluating its long-term efficacy in sustaining pain relief are lacking. Future research should investigate optimal music therapy protocols regarding session frequency, duration, and techniques required for maintaining benefits. Standardized measures over extended periods are needed to determine ideal regimens maximizing enduring outcomes.

Advances in neuroimaging, genetics, and machine learning techniques present opportunities to guide individualized music therapy based on a patient’s brain activity patterns, neurotransmitter function, and music preferences. Computational models could help determine optimal music parameters and predict treatment response. Personalized music selection and delivery may enhance clinical effects [267].

Caution should be taken regarding music characteristics like tempo, rhythm, melody, and volume [271]. Music with abruptly shifting tones or overly stimulating rhythms may exacerbate symptoms in certain chronic pain conditions. Sound levels should be carefully controlled to avoid adverse auditory effects. Overall, individualization of music therapy while considering safety factors can optimize its efficacy as a non-pharmacologic pain management approach.

### Others therapies

Additionally, some interventions not classified above also have widespread application in chronic pain management. These methods achieve analgesic effects by acting on different levels of the sensory modulation system.

#### Manual therapy and movement therapy

Manual therapy refers to hands-on techniques like joint mobilization and soft tissue manipulation aimed at restoring mobility and function [272]. Movement therapy utilizes specific exercises and physical training protocols to improve symptoms [273]. Both approaches influence neural mechanisms across peripheral, spinal, and central levels to alleviate chronic pain.

Manual therapy activates cutaneous mechanoreceptors and modulates spinal processing of sensory signals from the periphery [274]. Movement therapy increases BDNF and synaptic plasticity in pain modulatory circuits [275]. Both normalize imbalances in pro- and anti-inflammatory cytokines linked to chronic pain.

Recent evidence proposes manual therapy and movement therapy may prevent or reverse central sensitization through mechanisms like enhancing descending inhibition, correcting somatic dysfunctions, and optimizing sensory input patterns [276]. Neuroimaging shows normalized functional connectivity in chronic pain-related brain networks after therapy.

EEG and functional MRI have been utilized to evaluate responses and predict outcomes from manual and movement therapies [277]. Combining these approaches with pharmacological, psychological, and other interventions demonstrates enhanced pain relief through synergistic effects on physiological and psychological aspects of chronic pain.

Recent clinical trials demonstrate benefits of manual therapy and exercise for neuropathic pain, fibromyalgia, headache, and temporomandibular joint pain [272, 278–280] (Additional file 1: Fig. S4). The effects may involve modulating neural hypersensitivity, inflammation, and muscle dysfunction.

As adjuvants to NSAIDs, membrane stabilizers, and opioids, manual and movement therapies demonstrate enhanced analgesia compared to medication alone for low back pain, neck pain, and osteoarthritis, optimizing both physiological and cognitive-behavioral factors influencing chronic pain [281].

Integrating manual and movement therapies into psychological programs like CBT improves pain coping, catastrophizing, and kinesiophobia compared to either therapy alone [134]. Combination targets both physical dysfunction and maladaptive psychological factors in chronic pain.

While manual therapy and exercise demonstrate short-term pain relief, studies evaluating their long-term effectiveness and optimal protocols are lacking [282]. Future research should investigate the ideal number of sessions, treatment frequency, duration, and techniques required to obtain sustained benefits. Standardized measures over extended periods are needed to determine regimens that maximize enduring outcomes.

Safety considerations for manual therapy include screening for risk factors like osteoporosis and avoiding excessive forces [281]. For movement therapy, progression should be gradual and supervised to prevent overexertion. Special populations like elderly and postoperative patients require modified protocols to ensure safety and tolerability. Overall, individualization along with proper precautions can enhance the safe and effective application of these therapies.

#### Chiropractic care

Chiropractic care is a healthcare profession that uses manual therapy to treat structural and functional disorders of the spine and joints. Its therapeutic effect on pain relief involves influencing various neural mechanisms involved in pain modulation across different levels of the nervous system [283].

Chiropractic care employs manual techniques to treat musculoskeletal conditions and influence neural mechanisms involved in pain modulation. Chiropractic manipulation activates cutaneous mechanoreceptors and alters spinal processing of peripheral sensory inputs [284]. Emerging evidence suggests chiropractic increases BDNF, facilitating synaptic plasticity in pain pathways [285].

Chiropractic care may mitigate inflammation by reducing pro-inflammatory cytokines and improve circulation through somato-autonomic reflexes [286]. It is proposed to reverse central sensitization by correcting biomechanical and somatic dysfunctions influencing sensory input and motor output patterns.

Neuroimaging studies reveal chiropractic care normalizes aberrant activity and connectivity between chronic pain-related brain regions including the prefrontal cortex, anterior cingulate cortex, insula, and somatosensory cortex [287]. Electrophysiology demonstrates normalized neural hypersensitivity after treatment.

Recent clinical trials demonstrate benefits of chiropractic care for chronic pain conditions like fibromyalgia, headache, neuropathic pain, and temporomandibular disorders [288–291] (Additional file 1: Fig. S4). The effects may involve modulating neural hypersensitivity, somatic dysfunction, and muscle spasm.

Chiropractic combined with NSAIDs, muscle relaxants, and neuropathic agents demonstrates enhanced analgesia compared to medication alone for low back pain, neck pain, and extremity pain, optimizing the physical and neurological components [292].

Integrating chiropractic care into exercise and physical therapy programs results in improved outcomes including pain relief, range of motion, and function compared to individual therapies alone [293]. The combination targets multiple physiological and biomechanical factors underlying chronic musculoskeletal pain.

Emerging techniques like neuroimaging, genetics, and machine learning may enable individualized chiropractic treatment tailored to each patient’s characteristics and needs [294]. Biomarkers identifying joint dysfunction patterns could guide therapy. Real-time feedback systems could optimize force delivery and dosing for personalized care [294].

Safety considerations include screening for risk factors like osteoporosis, cord compression, and coagulopathies [295]. Excessive manipulation forces and frequencies should be avoided to prevent injury. Special populations like the elderly may require more gentle techniques. Guidelines on application and monitoring should be followed to prevent adverse events. Overall, an individualized approach with proper precaution enhances chiropractic treatment safety and efficacy.

#### Biofeedback

Biofeedback is a non-invasive technique that uses sensors to measure physiological signals such as heart rate, muscle tension, skin temperature, or brain activity and provides real-time feedback to help individuals develop awareness and control over these signals [296]. When combined with psychosocial or physical therapies, biofeedback may help relieve chronic pain by altering bodily functions and influencing pain-related brain networks [297, 298].

It should be noted that biofeedback is rarely used as a standalone intervention, but rather integrated with other physical and psychological therapies. The mechanisms of biofeedback should be understood in light of its synergistic effects when combined with modalities like exercise, massage, CBT, and more. Focusing solely on the mechanisms of biofeedback itself risks decontextualizing it from real-world, multimodal applications.

Integrating biofeedback into exercise, massage, and physical therapy programs results in greater improvements in pain, mobility, and quality of life versus either therapy alone for musculoskeletal pain, temporomandibular joint disorders, irritable bowel syndrome, chronic fatigue syndrome, and pelvic pain [299–304] (Additional file 1: Fig. S4).

Combining biofeedback with CBT, mindfulness, and hypnosis therapy shows greater reductions in pain-related anxiety, fear avoidance, and catastrophizing thoughts compared to individual treatments [305]. Multimodal approaches utilizing biofeedback may achieve optimal pain self-regulation.

Caution should be taken with electrode placement and stimulus intensity during biofeedback. Patients with cardiac devices or seizures require modified protocols [306]. Guidance on use and monitoring should be provided, especially for home-based treatment. Combining self-regulation techniques with biofeedback may optimize skills retention and adherence.

Future studies could examine innovative biofeedback modalities, remote delivery methods, and integration with VR or gaming for engagement. Big data analytics and AI could uncover new strategies to enhance pain self-management through biofeedback [307]. Overall, advancing biofeedback technology while ensuring appropriate applications can maximize its potential as a chronic pain treatment.

#### VR therapy

VR therapy utilizes computer-generated environments to create immersive experiences for chronic pain relief [308]. VR therapy may achieve analgesia through multiple mechanisms, including cognitively distractive effects, visuotactile stimulation, and visuomotor stimulation [309, 310]. These multimodal effects may induce neuroplastic changes by altering sensory perception and modulating pain-related neural processing. Studies suggest that VR therapy can induce analgesia, modulate neural plasticity, and alter brain activity related to anxiety disorders.

Emerging evidence suggests VR therapy may prevent or reverse central sensitization, a key mechanism in the transition from acute to chronic pain [311]. At the molecular level, VR therapy reduces NMDA receptor activation and neuropeptide release involved in spinal cord hyperexcitability based on animal studies. It normalizes elevated COX-2, TNF-α, and IL-1β levels associated with neuroinflammation in pain pathways [312]. At the cellular level, VR therapy decreases dorsal horn neuron firing and wind-up in response to repeated stimuli. It suppresses long-term potentiation of synaptic transmission between nociceptive afferents and spinal projection neurons [313]. These effects indicate VR therapy may inhibit the molecular and cellular processes driving central sensitization. Clinically, VR applied early after injury prevents chronic pain development compared to conventional treatment in human studies. Overall, VR therapy holds promise for inhibiting maladaptive neuroplasticity underlying the progression from acute to chronic pain through counteracting the key mechanisms of central sensitization.

Neuroimaging reveals VR therapy activates endogenous anti-nociception systems like the periaqueductal gray and nucleus raphe magnus, which engage descending inhibition of nociceptive signals [314]. Functional MRI shows VR therapy enhances functional connectivity between periaqueductal gray and pain-processing cortical regions to strengthen top-down pain modulation [315].

VR therapy facilitates neuroplastic changes in brain networks involved in sensory, affective and cognitive processing of chronic pain. Resting-state functional MRI indicates VR normalizes aberrant default mode network connectivity associated with clinical pain [315]. Diffusion MRI shows VR alters structural connectivity between thalamus, periaqueductal gray, and sensory cortices critical for pain transmission [316].

Recent clinical trials demonstrate VR analgesic effects in diverse chronic pain populations beyond neuropathic and postoperative pain. In fibromyalgia, VR reduces pain scores and increases threshold compared to control treatment [317]. For migraine, VR adjunctive to pharmacologic prophylaxis leads to greater reductions in headache frequency and disability than drug therapy alone [318]. In cancer pain, VR combined with opioids provides superior analgesia at lower opioid doses versus opioids alone, highlighting the broad applicability of VR across different chronic pain conditions [319] ( Additional file 1: Fig. S4).

Importantly, research shows VR has synergistic benefits when combined with pharmacological and non-pharmacological treatments. For example, VR plus pregabalin reduces neuropathic pain and pregabalin-associated somnolence more than either treatment alone [320]. VR integrated into physiotherapy yields greater improvements in function compared to individual therapies for chronic low back pain [321]. VR adjunctive to CBT enhances pain coping and reduces anxiety better than either therapy alone [322]. Such combinatorial approaches facilitate multimodal engagement of sensory, cognitive, and emotional aspects to optimize outcomes. Overall, these studies highlight VR as a promising adjuvant that can amplify analgesic effects within multidisciplinary chronic pain care.

While VR therapy shows promise, there are limitations needing consideration. Individual differences in immersion and side effects like cybersickness can influence treatment response [323]. For patients with allodynia or hyperalgesia, visuotactile and visuomotor content requires caution as it may exacerbate symptoms [324]. In these cases, individualized approaches avoiding tactile or motor stimulation may be necessary. Moving forward, advances in VR technology and machine learning could enhance personalization and accessibility. For example, AI and neuroimaging may reveal biomarkers to optimize protocols on an individualized basis. Multisensory simulations tailored to specific pain conditions and customized triggers could improve therapeutic benefits. Through human-centered design and integrating engineering innovations with neuroscience insights, VR therapy can achieve its full potential as an effective personalized treatment for chronic pain.

#### Non-invasive brain stimulation (NIBS)

NIBS utilizes magnetic or electric fields to modulate brain activity for pain relief. Different NIBS techniques have different mechanisms of action on pain-related neural circuits [325]. It is postulated that NIBS such as transcranial magnetic stimulation (TMS) and transcranial direct current stimulation (tDCS) modulates cortical excitability and activity to induce analgesia.

Spectroscopy studies show NIBS alters levels of neurotransmitters including glutamate, GABA, serotonin, and dopamine critical for pain processing [325]. Proteomics analysis indicates NIBS modulates expression of receptors like NMDA, opioid, and TRPV1 involved in nociception [326].

Neuroimaging studies reveal the molecular effects of NIBS manifest as changes in pain-related neural circuitry [327]. PET imaging indicates NIBS engages descending pain inhibitory pathways including the periaqueductal gray, rostral ventromedial medulla, and opioidergic nucleus cuneiformis [328]. Functional MRI reveals NIBS normalizes aberrant activity and connectivity in the default mode network, salience network, and sensorimotor network which underlie cognitive, affective, and sensory dimensions of chronic pain [329].

NIBS facilitates neuroplasticity through long-term potentiation and depression of synaptic strength. Structural MRI shows NIBS induces gray matter changes in pain-related regions including thalamus, somatosensory cortex, anterior cingulate cortex, and insula [330].

Emerging NIBS techniques like temporal interference stimulation, transcranial pulsed ultrasound, and transcutaneous vagus nerve stimulation target peripheral nerves and the vagus nerve to engage endogenous analgesia mechanisms [331, 332]. Temporal interference stimulation works by delivering out-of-phase currents to the head which affects pain-related cortical areas [333]. Transcranial pulsed ultrasound modulates brain circuits through mechanical and thermal effects [331]. Vagus nerve stimulation activates the vagus nerve’s connection to the locus coeruleus and pain modulatory nuclei [332]. These novel techniques expand the scope of NIBS for non-invasive pain relief.

Recent studies demonstrate analgesic effects of NIBS in diverse chronic pain populations beyond neuropathic pain and headaches, including cancer-related pain, visceral pain, abdominal pain, fibromyalgia, and musculoskeletal pain [334–338] (Additional file 1: Fig. S4). NIBS also reduces acute postoperative pain and opioid consumption. These findings highlight the wide applicability of NIBS for pain management.

Importantly, NIBS demonstrates enhanced pain relief when combined with pharmacological and non-pharmacological therapies. For instance, NIBS applied before motor cortex stimulation improves treatment outcomes for neuropathic pain. NIBS combined with exercise therapy yields greater gains in pain and function compared to either therapy alone for fibromyalgia [339]. This highlights NIBS as a promising adjuvant that can amplify analgesic effects and improve clinical outcomes when combined with other treatment modalities as part of multidisciplinary care.

While promising, there are limitations of NIBS warranting consideration. The mechanisms underlying different techniques remain incompletely understood [340]. Response variability between patients and unpredictable effects over time require further study. Safety and tolerability depend on stimulus parameters and individual factors. Headaches, fatigue, nausea, insomnia, and pain exacerbation can sometimes occur. Special consideration is required for vulnerable populations like pediatric, elderly, and pregnant patients.

Moving forward, advances in computational modeling and machine learning applied to neuroimaging, electrophysiological, genetic, and clinical data could help optimize personalized NIBS protocols tailored to each patient’s symptoms, biomarkers, and responses. Technological improvements such as novel electrode designs, wearable devices, and integration with function-triggered feedback systems could enhance individualized delivery, safety, and outcomes. Development of NIBS protocols adapted for home use with telemonitoring may increase accessibility. Further research on combination strategies with emerging treatments like immunomodulation, VR therapy, and psychedelics could uncover synergies [341]. Overall, continued innovation and optimization of NIBS technology and protocols through a precision medicine approach will help fulfill its potential as a safe, effective personalized therapy for diverse chronic pain disorders.

## Summary: common mechanisms of major NINPT modalities

Now that we have reviewed the mechanisms of major NINPT modalities respectively, it is worth summarizing some of their common principles from a comparative perspective. While NINPT modalities differ in their techniques, they share some common mechanisms that confer analgesic effects:

- *Modulating nociceptive signaling—*Most NINPT can inhibit pain signal transmission in the peripheral and central nervous system through effects on ion channels, neurotransmitters, and inflammation.
- *Enhancing descending modulation—*Many NINPT engage endogenous analgesic pathways involving opioids, serotonin, and norepinephrine to exert inhibitory effects.
- *Influencing psychology and emotions—*NINPT like CBT and hypnosis target affective and cognitive dimensions of pain.
- *Promoting neuroplasticity—*Some NINPT may facilitate neuroplastic changes in pain pathways through mechanisms like long-term potentiation (LTP)/long-term depression (LTD).
- *Limiting sensitization—*Certain NINPT are proposed to prevent or reverse central sensitization underlying chronic pain.

Although the specific molecular and cellular effects vary across techniques, summarizing these common principles may provide a preliminary framework to guide optimal application and combination of different NINPT modalities. Despite current differences, the common mechanisms of major NINPT modalities highlighted here represent our initial attempts to inform the selection and integration of appropriate techniques for individualized, multimodal chronic pain management.

## Limitations and challenges in the use of NINPT

While NINPT demonstrates potential for managing pain without medications or invasive procedures, there are limitations warranting consideration.

### Heterogeneity and individualization

The heterogeneity of chronic pain conditions and individual variability in treatment response highlight the need to tailor NINPT protocols based on each patient’s specific needs and mechanisms [342–344]. Treatment outcomes should be monitored and adjusted accordingly. Advances in predictive biomarkers could guide individualized approaches.

### Standardization

The lack of standardized treatment protocols and dosing guidelines makes it difficult to compare efficacy and generalize findings across different NINPT modalities and trials [345, 346]. Validated, reliable outcome measures are needed to quantify neural activity changes and pain relief. Computational modeling and machine learning may help optimize protocols.

### Accessibility and adherence

Practical barriers like affordability, insurance coverage, and access to specialized equipment or facilities may limit utilization of certain NINPT. Patient adherence may suffer if expectations are not properly managed. Cost-effectiveness studies and enhanced engagement strategies are required to promote adherence and accessibility [347].

### Research methodology limitations

Current studies exhibit issues like small sample sizes, inadequate randomization, blinding difficulties, and lack of long-term follow-up. Research gaps exist in elucidating mechanisms and predictive biomarkers. Pragmatic comparative trials of different NINPT protocols are needed. Addressing these limitations through rigorous research is key to inform clinical translation and optimize patient outcomes [348].

## Future outlook and recommendations

To realize the full potential of NINPT, further innovation and optimization is needed across several fronts:

### Advancing personalized and precision therapies

Emerging techniques utilizing computational modeling, neuroimaging, omics, and machine learning have proposed personalized medicine approaches for chronic pain treatment [349–351]. Further integration of these technologies could facilitate developing individualized NINPT protocols tailored to each patient’s specific symptoms, predictive biomarkers, and treatment responses. Additionally, combining NINPT with nutrition and lifestyle interventions may also help optimize therapeutic outcomes.

### Promoting integrative and combinatorial treatments

Synergistic combination strategies that incorporate NINPT modalities with pharmacological, psychological, physical, and complementary interventions are investigated through a systems approach [352, 353]. This multimodal integration can better engage pain mechanisms at peripheral, spinal, and supraspinal levels.

### Harnessing emerging technologies and neuroscience insights

Applying biosensors, wearables, and implantables enables adaptive closed-loop NINPT delivery [354, 355]. Utilizing advanced techniques like optogenetics, holographic microscopy, and functional neuroimaging to elucidate NINPT mechanisms across multiple levels of analysis.

### Improving research methodology

Addressing limitations in study design, standardization, comparator selection, and long-term assessments through pragmatic, large-scale comparative trials focused on patient-centered outcomes [356, 357]. This will strengthen the evidence base to inform guidelines for clinically translating different NINPT.

## Conclusions

**Fig. 4:**
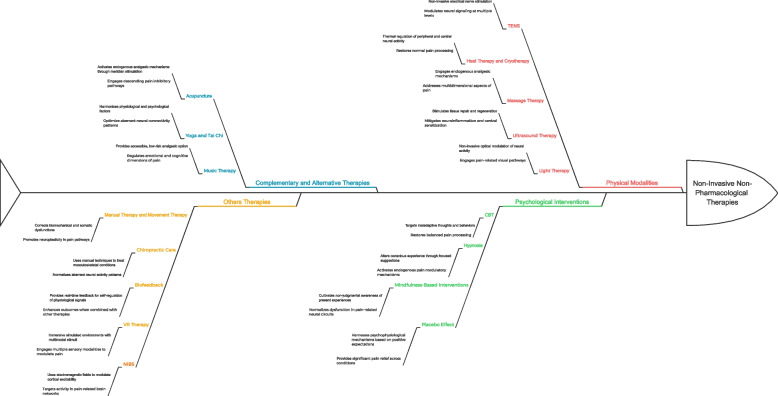
Overview of non-pharmacological therapies for chronic pain. Note: TENS: transcutaneous electrical nerve stimulation; CBT: cognitive-behavioral therapy; VR: virtual reality

NINPT represents a promising domain encompassing diverse interventions that modulate pain through peripheral, spinal, and supraspinal mechanisms. This review synthesizes current evidence on mechanisms and clinical potential of major NINPT modalities. While results are promising, limitations warrant attention (Fig. 4).

Realizing the full benefits of NINPT requires a personalized, integrative approach combining technologies, research innovations, and therapeutic strategies across pharmacological, psychological, and physical domains. Advancing NINPT through translational research and addressing methodology limitations will provide the evidence base to inform clinical guidelines and integrate NINPT into precision care models.

Overall, this review bridges the gap between neuroscience insights and clinical translation to highlight the value of NINPT as a complementary non-pharmacological option ready to be tailored to individual needs and integrated into contemporary pain medicine. We hope this mechanistic framework promotes implementation of NINPT in clinical practice to ultimately improve outcomes and quality of life for chronic pain patients.

### Supplementary Information

**Additional file 1: Figure S1.** Clinical applications of physical modalities for chronic pain conditions. Note: Chronic pain conditions in the figure refer to ICD-11 classifications. Examples given in the article include specific diseases falling into these categories. **Figure S2.** Clinical applications of psychological interventions for chronic pain conditions. Note: Chronic pain conditions in the figure refer to ICD-11 classifications. Examples given in the article include specific diseases falling into these categories. **Figure S3.** Clinical applications of complementary and alternative therapies for chronic pain conditions. Note: Chronic pain conditions in the figure refer to ICD-11 classifications. Examples given in the article include specific diseases falling into these categories. **Figure S4.** Clinical applications of others therapies for chronic pain conditions. Note: Chronic pain conditions in the figure refer to ICD-11 classifications. Examples given in the article include specific diseases falling into these categories.

## Data Availability

Not applicable.

## Abbreviations

ACC: Anterior cingulate cortex
BDNF: Brain-derived neurotrophic factor
CBT: Cognitive-behavioral therapy
COMT: Catechol-o-methyltransferase
COX-2: Cyclooxygenase-2
EEG: Electroencephalogram
IC: Insula cortex
IL-6: Interleukin-6
MRI: Magnetic resonance imaging
NIBS: Non-invasive brain stimulation
NINPT: Non-invasive non-pharmacological therapies
NSAIDs: Nonsteroidal anti-inflammatory drugs
PAG: Periaqueductal gray
PCR: Polymerase chain reaction
PET: Positron emission tomography
PFC: Prefrontal cortex
RVM: Rostral ventromedial medulla
tDCS: Transcranial direct current stimulation
TENS: Transcutaneous electrical nerve stimulation
TMS: Transcranial magnetic stimulation
TNF-α: Tumor necrosis factor alpha
VR: Virtual reality
