# Supplementary material for: Multimodal non-invasive non-pharmacological therapies for chronic pain: mechanisms and progress
Source: BMC Med. 2023 Sep 29;21:372. doi: 10.1186/s12916-023-03076-2 (PMC10542257; doi:10.1186/s12916-023-03076-2)
Supplement: Supplementary file 1 — Additional file 1: Figure S1. Clinical applications of physical modalities for chronic pain conditions. Note: Chronic pain conditions in the figure refer to ICD-11 classifications. Examples given in the article include specific diseases falling into these categories. Figure S2. Clinical applications of psychological interventions for chronic pain conditions. Note: Chronic pain conditions in the figure refer to ICD-11 classifications. Examples given in the article include specific diseases falling into these categories. Figure S3. Clinical applications of complementary and alternative therapies for chronic pain conditions. Note: Chronic pain conditions in the figure refer to ICD-11 classifications. Examples given in the article include specific diseases falling into these categories. Figure S4. Clinical applications of others therapies for chronic pain conditions. Note: Chronic pain conditions in the figure refer to ICD-11 classifications. Examples given in the article include specific diseases falling into these categories. [file 12916_2023_3076_MOESM1_ESM.docx]

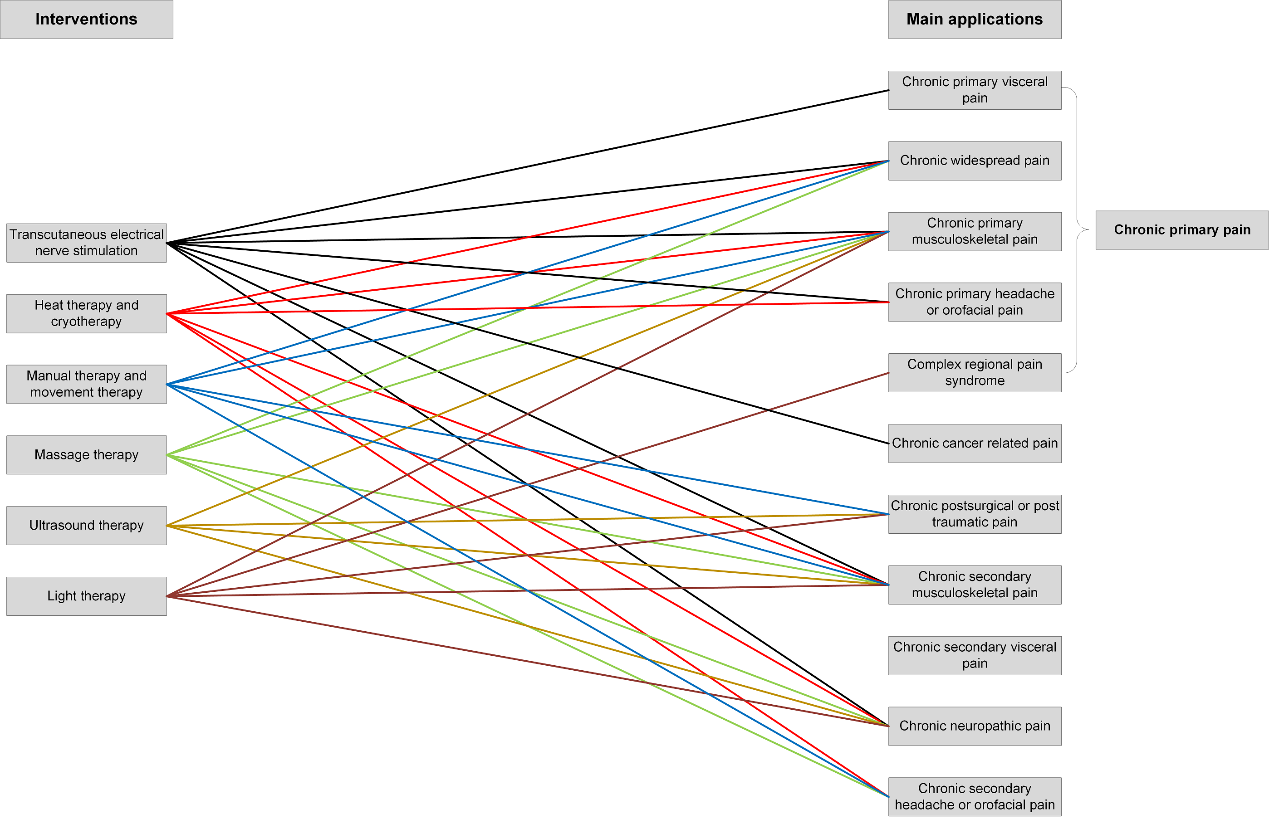


**Figure S1.** Clinical applications of physical modalities for chronic pain conditions.

**Note:** Chronic pain conditions in the figure refer to ICD-11 classifications. Examples given in the article include specific diseases falling into these categories.


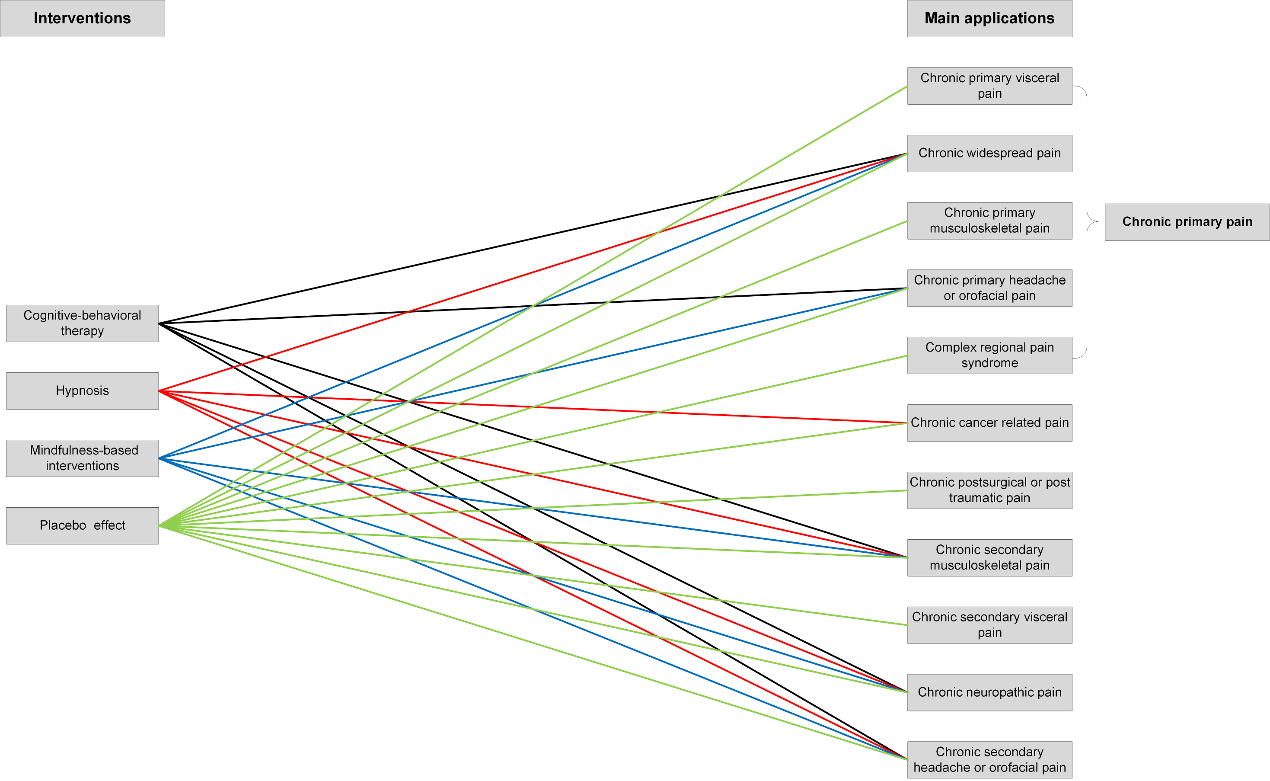


**Figure S2.** Clinical applications of psychological interventions for chronic pain conditions.

**Note:** Chronic pain conditions in the figure refer to ICD-11 classifications. Examples given in the article include specific diseases falling into these categories.


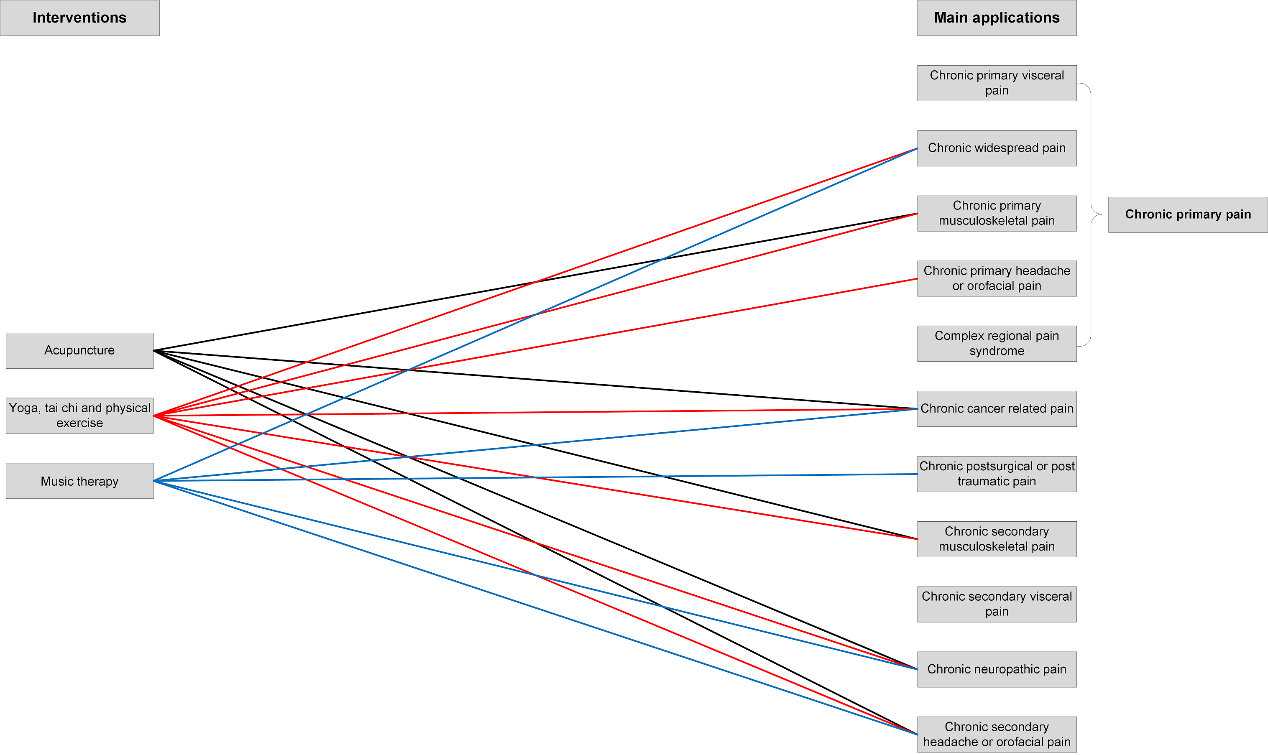


**Figure S3.** Clinical applications of complementary and alternative therapies for chronic pain conditions.

**Note:** Chronic pain conditions in the figure refer to ICD-11 classifications. Examples given in the article include specific diseases falling into these categories.


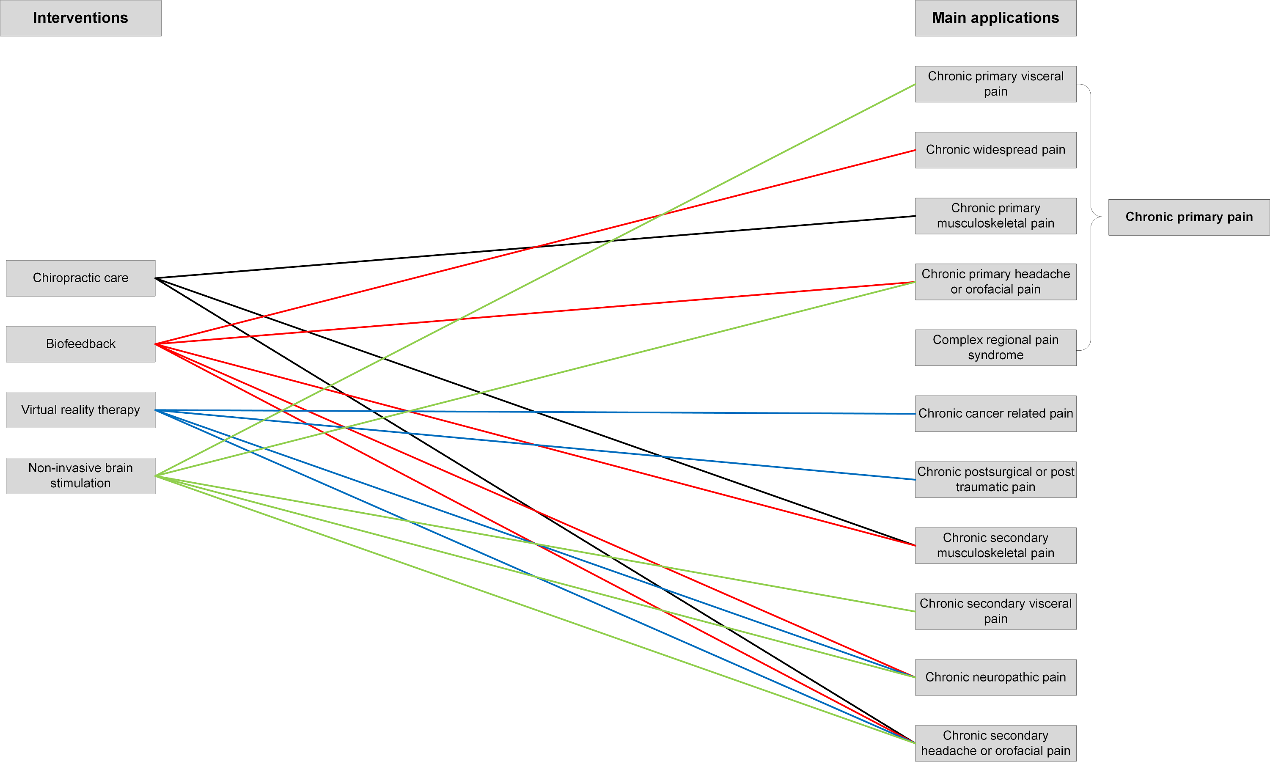


**Figure S4.** Clinical applications of others therapies for chronic pain conditions.

**Note:** Chronic pain conditions in the figure refer to ICD-11 classifications. Examples given in the article include specific diseases falling into these categories.
